# Test–Retest Reliability of Synchrony and Metastability in Resting State fMRI

**DOI:** 10.3390/brainsci12010066

**Published:** 2021-12-31

**Authors:** Lan Yang, Jing Wei, Ying Li, Bin Wang, Hao Guo, Yanli Yang, Jie Xiang

**Affiliations:** College of Information and Computer, Taiyuan University of Technology, Taiyuan 030024, China; yanglan0333@link.tyut.edu.cn (L.Y.); 20141032@sxufe.edu.cn (J.W.); liying01@tyut.edu.cn (Y.L.); wangbin01@tyut.edu.cn (B.W.); guohao@tyut.edu.cn (H.G.); yangyanli@tyut.edu.cn (Y.Y.)

**Keywords:** synchrony, metastability, test–retest reliability, resting-state network, resting state fMRI

## Abstract

In recent years, interest has been growing in dynamic characteristic of brain signals from resting-state functional magnetic resonance imaging (rs-fMRI). Synchrony and metastability, as neurodynamic indexes, are considered as one of methods for analyzing dynamic characteristics. Although much research has studied the analysis of neurodynamic indices, few have investigated its reliability. In this paper, the datasets from the Human Connectome Project have been used to explore the test–retest reliabilities of synchrony and metastability from multiple angles through intra-class correlation (ICC). The results showed that both of these indexes had fair test–retest reliability, but they are strongly affected by the field strength, the spatial resolution, and scanning interval, less affected by the temporal resolution. Denoising processing can help improve their ICC values. In addition, the reliability of neurodynamic indexes was affected by the node definition strategy, but these effects were not apparent. In particular, by comparing the test–retest reliability of different resting-state networks, we found that synchrony of different networks was basically stable, but the metastability varied considerably. Among these, DMN and LIM had a relatively higher test–retest reliability of metastability than other networks. This paper provides a methodological reference for exploring the brain dynamic neural activity by using synchrony and metastability in fMRI signals.

## 1. Introduction

The brain is a complex nonlinear dynamic system, and the neural oscillations generated by individual neurons or the interaction between neurons define different cognitive and behavioral states [1,2,3,4,5]. Nonlinear system has two important dynamic characteristics--synchronization and metastability [6,7], which play very important roles [8,9,10,11,12]. Neuronal synchrony plays a role in well-timed coordination and communication between neural populations simultaneously engaged in a cognitive process [13], and metastability reflects flexible dynamic interactions between neural populations [14]. Specific to the brain network, synchrony in the oscillatory activity of network regions is considered to underpin information exchange [15], whereas metastability represents the variability in the synchronization of network regions over time that is considered important for adaptive information processing [16,17,18], and can be estimated by calculating the well-defined order parameter [19,20,21,22,23]. Existent theories suggest that synchrony is considered as the core mechanism for sculpting communication and plasticity of the entire brain network that underpins human cognition [24], and metastability can reconcile the competing demands of integration and segregation of brain regions interact [17,25,26].

In recent years, there has been increasing research on the measurement of synchrony and metastability in functional magnetic resonance imaging (fMRI) signals [25,27,28,29,30]. Additionally, it is well-verified by a variety of studies that these indexes not only provide a mechanistic explanation of the origin of functional organization of the brain [28,31,32,33,34], but also help us understand the mechanistic causes of diseases [18,25,35], and as a significant predictor of diseases. For example, a study conducted by Alderson et al. examined the causal link between damage in high participation nodes, reduced metastability of neural dynamics, and decline in global cognitive performance [25]. Moreover, there is also a published study that describes that some of these changes in metastability are associated with the suppression of a given network during task completion [26]. Moreover, a recent study by Naik et al. assesses the changes in metastability to characterize age-effects on the dynamic repertoire of the functional networks at rest [29]. In a word, these two neurodynamic indexes facilitate the exploration of a larger dynamical repertoire of the brain and allow for the all-around visitation of functional states and dynamic responses to the external world [36].

Although particular attention is paid to synchrony and metastability, only a few studies have systematically analyzed the test–retest reliability of them when applied to fMRI signals. At present, there is evidence that any variable or factor with significant intra- or inter-individual variability can influence test–retest reliability [37]. According to studies of data acquisition, different field strengths have different blood oxygenation level-dependent (BOLD) contrast [38,39,40,41,42,43]. Theoretically, 3T fMRI offers twice the signal of 1.5T fMRI, and the greater sensitivity in the detection of signal changes, increased signal-to-noise ratio (SNR), BOLD signal change, and BOLD contrast-to-noise ratio (CNR) [44]. Therefore, questions have been raised regarding whether different field strengths affect the test–retest reliability of synchrony and metastability in the fMRI signal. Additionally, previous studies have argued that the shorter repetition times (TR), the higher temporal resolution, and the more time points, which has a higher statistical power. Moreover, high temporal resolution increases BOLD sensitivity [45,46], so will the temporal resolution affect the test–retest reliability of synchrony and metastability? The high spatial resolution would yield a marked increase in functional contrast relative to low resolution [47], so how about the effect of reliability of synchrony and metastability? Meanwhile, measures of fMRI are dynamic, and may be subject to modulations related to an individual’s current state. Further analysis revealed that test–retest reliabilities of network metrics were sensitive to scanning intervals, repeated measurements taken over shorter intervals are more reliable than those taken over longer intervals [48,49,50,51,52,53], is this also true for neurodynamic indexes? In addition, apart from these different parameter settings during data collection, various types of noise and artifacts in the fMRI data collection process would pollute data [54,55,56,57]. Therefore, will the reliability of these two indexes be improved after denoising? In particular, what about the stability of neurodynamic indexes in different resting-state networks? Therefore, factors that may affect the dynamic changes in the functional connection also raise concerns about the test–retest reliabilities of these two indexes.

In this study, the test–retest performance of synchrony and metastability was analyzed. We used the resting-state functional magnetic resonance imaging (rs-fMRI) datasets from healthy young people to calculate and analyze the mentioned factors to determine whether they have an impact on neurodynamic indexes. On this basis, we also measure the test–retest reliabilities of synchrony and metastability in the resting-state network, to discover more networks characteristic. Our research aims to provide a reference for researchers who would use synchrony and metastability in fMRI signals.

## 2. Materials and Methods

**Figure 1:**
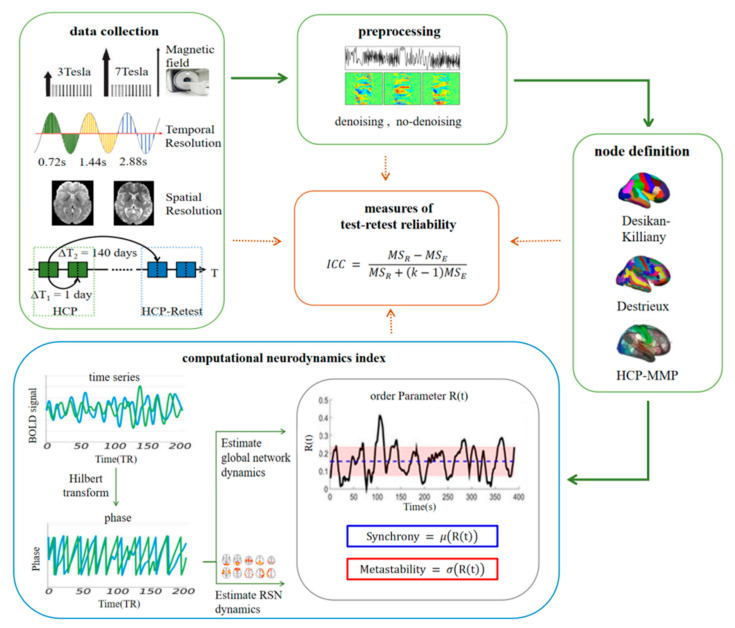
Overview of the workflow. Green boxes represent different factors. The orange box and blue box represent ICC index calculation and neurodynamic indexes calculation, respectively. ∆T1 can be used to evaluate short-term reliability and ∆T2 evaluates long-term reliability. See the methods for details.

An overview of the workflow is shown in Figure 1. To examine the stability of synchrony and metastability in the fMRI signals, and to analyze the influences of different factors on test–retest reliability (the green boxes in Figure 1), we extracted the mean time series for each node from the preprocessed data of all subjects, subsequently calculated synchrony and metastability in the global network and different resting-state networks, and their stabilities were measured.

### 2.1. Datasets

Given the multi-parameter conditions required for studies, more diverse fMRI datasets from the Human Connectome Project (HCP) 1200 Subjects Release (S1200) were used [58], including 3T rs-fMRI data (3T), 3T rs-fMRI ICA-FIX cleaned data (3T-FIX), 7T rs-fMRI ICA-FIX cleaned data (7T-FIX) and 3T rs-fMRI retest data (HCP_Retest).

Both 3T and 3T-FIX data were scanned with a Siemens Prisma 3 Tesla scanner. Their fMRI data were acquired using a gradient-echo EPI sequence with the following parameters: TR = 720 ms, TE = 33.1 ms, flip angle = 52°, field of view = 208 × 180 mm^2^, spatial resolution = 2 × 2 × 2 mm^3^. 7T-FIX data were scanned with a Siemens Prisma 7 Tesla scanner. The fMRI data were acquired using a gradient-echo EPI sequence with the following parameters: TR = 1000 ms, TE = 22.2 ms, flip angle = 45°, field of view = 208 × 208 mm^2^, spatial resolution = 1 × 1 × 1 mm^3^.

Each subject underwent four rs-fMRI runs of approximately 14.4 min each (1200 images): two in the first session (Day 1) and two in the second session (Day 2). After 140 days, four other rs-fMRI runs of data for 45 subjects were collected in the same way. Since there were 44 subjects who were the same in the two collections, and three of them lacked information for rs-fMRI scans, we chose 41 subjects for our test–retest study. However, only 22 subjects were the same in the two collected data of 7T-FIX, so when analyzing the effect of magnetic flux strength strategies, 169 common subjects of all 3T-FIX and 7T-FIX were used for short-term retest analysis. This study includes four rs-fMRI runs of data for 169 subjects and eight rs-fMRI runs of data for 41 subjects. Among them, the mean interval between scan 1 and 3, scan 2 and 4, scan 5 and 7, scan 6 and 8 was 1 day, and the mean interval between scan 1 and 5, scan 2 and 6, scan 3 and 7, scan 4 and 8 was 140 days (Figure 1).

In order to explore the effect of spatial resolution on reliabilities of synchrony and metastability, we also used the Institute of Psychology, Chinese Academy of Sciences dataset (IPCAS) here. During the rest scan, a fixation cross was presented to the first group of 29 subjects, and the subjects were instructed to rest while focusing on the fixation cross. Four resting-state scans were obtained for each subject using a Siemens 3T scanner. Researchers acquired the echo-planar imaging (EPI) functional volumes of each scan (time repetition (TR) = 2000 ms; time echo (TE) = 30 ms; flip angle (FA) = 90 μ, number of slices = 32, matrix = 64 × 64; field of view (FOV) = 256 mm, spatial resolution = 4 × 4 × 4 mm^3^) and structural MRI data using sagittal T1-weighted magnetization prepared rapid gradient echo (MPRAGE) sequences (TR = 2530 ms; TE = 2.51 ms; inversion time = 1100 ms; FA = 7 μ; number of slices = 128; FOV = 256 mm). The mean interval between scan 1 and 2, and scan 3 and 4 was 29 min, and the mean interval between scan 1 and 3, and scan 2 and 4 was one week. The preprocessing method we use is consistent with that of the HCP dataset.

### 2.2. Data Preprocessing

Data preprocessing and quality control (including head motion) were implemented through the HCP pipeline [59]. The fMRI pipelines include following steps: distortion correction, motion correction, registration in structural data, and conversion to gray-ordinates standard space [60]. Meanwhile, the “ICA-FIX” denoised fMRI data of the HCP subjects were used, which were processed by using an automatic denoising approach based on independent component analysis (ICA) followed by FMRIB’s ICA-based X-noiseifier to minimize head motion by removing structured artifacts [61,62]. Each subject’s preprocessed fMRI data were resampled to a common standard cortical surface mesh representation (fs_LR 32 k mesh) [63]. Then, the average value of the vertex strength within each region based on the brain atlas was extracted to obtain the time series. At the same time, we also downsampled the datasets to different repetition times (TR) settings from TR 0.72 s to 1.44 s and 2.88 s, respectively, by taking every n-th [n = 2,4] sample from every time series.

### 2.3. Dynamical Metrics

We used the extracted time series of each participant, and applied them with the Hilbert transform to calculate the associated analytical signals. In order to assess measurements of network dynamics within the brain, we evaluated the Kuramoto order parameters $R\left(t\right)$, which was estimated for (1), the set of a region comprising whole-brain network (2), the set of regions comprising single resting-state network, and (3), when evaluating their interactions, the set of regions comprising two resting-state networks, defined by:

(1) $$R\left(t\right)\;=\frac{1}{N}\left|\sum_{n=1}^{N}e^{{i\varphi }_{n}\left(t\right)}\right|,$$

where N is the number of brain regions and $\varphi_{n}\left(t\right)$ is the instantaneous phase of regional mean BOLD time series in region n. We considered the mean of the order parameter $R\left(t\right)$ across time, as an index of synchrony and the standard deviation of the $R\left(t\right)$, as an index of metastability [28]. After calculation, we got the global measurement value of synchrony and metastability, the measurement value of resting-state networks, and the interaction matrix between resting-state networks.

### 2.4. Node Definition

In this study, we adopted three widely used functional parcellations. The Desikan-Killiany atlas was an automated labeling system for subdividing the human cerebral cortex on magnetic resonance imaging (MRI) scans into 68 gyral-based regions of interest [64]. Additionally, the Destrieux atlas has the same principles as the Desikan-Killiany atlas, producing a cortical parcellation with 148 independent sulcal and gyral regions [25,65]. Another cortical areal parcellation (HCP-MMP atlas) contains 360 distinct areas, symmetrically arranged across the two hemispheres [66]. In addition, we also divided multiple resting-state networks (RSNs) for the Destrieux atlases. We specifically examined the default mode network (DMN), the limbic network (LIM), the frontoparietal control network (FPN), the somatomotor network (SMN), the ventral attention network (VAN), the dorsal attention network (DAN), and the visual network (VIS).

### 2.5. Test–Retest Reliability

The test–retest reliability evaluates the statistical stability of the index at different measurement times, and the intraclass correlation coefficient (ICC) is considered a frequently used reliability coefficient index to measure it [67]. It does not just comprehensively consider the changes within the individual and among different individuals, but also reflects the stability and consistency of the index over time [68]. The ICC value can be calculated according to the following formula:

(2) $$\mathrm{ICC}=\frac{{\mathrm{MS}}_{R}-{\mathrm{MS}}_{E}}{{\mathrm{MS}}_{R}\;+\left(k-1\right){\mathrm{MS}}_{E}}$$

${\mathrm{MS}}_{R}$ represents the mean square between subjects, ${\mathrm{MS}}_{E}$ represents the residual mean square, and k is the number of repeated observations per subject.

In this study, ICC values were usually divided into five common intervals: 0 < ICC ≤ 0.25 indicated poor reliability; 0.25 < ICC ≤ 0.4 indicated low reliability; 0.4 < ICC ≤ 0.6 indicated fair reliability; 0.6 < ICC ≤ 0.75 showed that reliability was good; and 0.75 < ICC ≤ 1.0 meant that reliability was very good, close to perfect. In practice, we usually expect to have a fair to almost perfect reliability index (ICC > 0.4) [52]. In this study, we specified that scans with short intervals (1 day) were used to calculate short-term reliability, and scans with long intervals (140 days) were used to calculate long-term reliability.

### 2.6. Statistical Analysis

We have measured the reliability of synchrony and metastability on both the global and resting-state networks. As the ICC is already a statistical indicator, our further statistical analysis could only be performed based on the RSNs. Other statistical analyses were performed using the statistic toolbox SPSS 19. To further explore significant differences, we calculated the paired sample *t*-test in reliability of synchrony and metastability among different factor analysis on the RSNs. The experimental results are included in the Supplementary Materials.

## 3. Results

### 3.1. Effects of Different Magnetic Flux Strength Strategies on Reliabilities of Synchrony and Metastability

**Figure 2:**
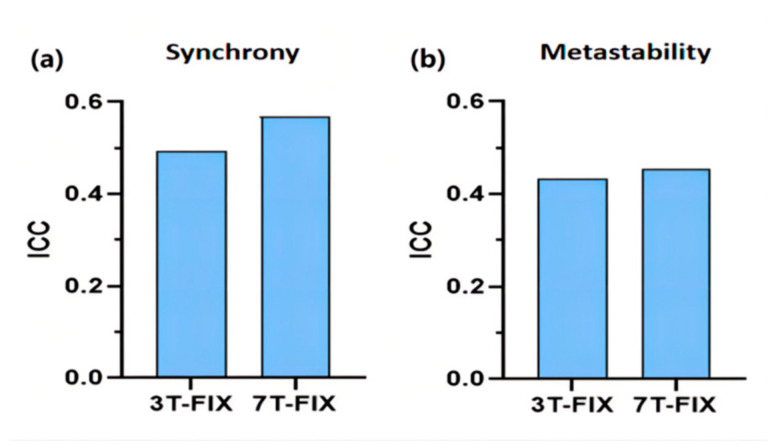
Reliabilities of synchrony (**a**) and metastability (**b**) in different magnetic flux strength strategies, evaluated by the intraclass correlation coefficient (ICC).

We validated the influence of different magnetic flux strengths on them by calculating the test–retest reliability analysis of synchrony and metastability with denoised fMRI data divided according to the Destrieux atlas. As shown in Figure 2, the retest reliability of synchrony of 7T-FIX was higher than that of 3T-FIX. Notably, they all showed fair reliability (mean ICC > 0.4). Due to the small amount of retest data for 7T, all of the follow-up studies used 3T datasets for analysis.

### 3.2. Effects of Different Temporal Resolution Strategies on Reliabilities of Synchrony and Metastability

**Figure 3:**
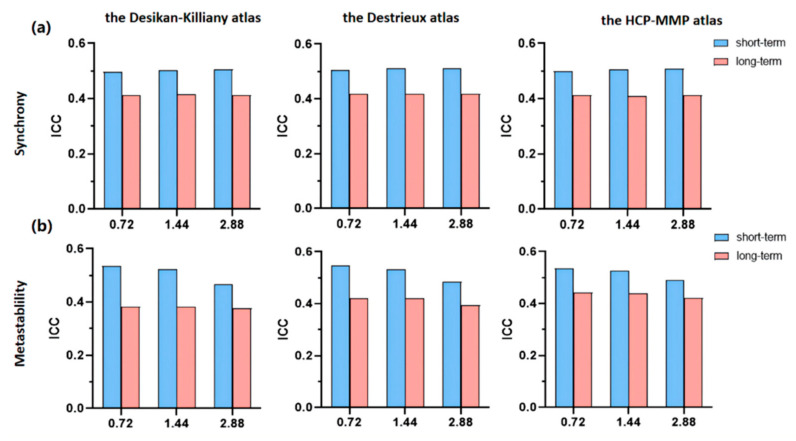
Reliabilities of synchrony (**a**) and metastability (**b**) in different temporal resolutions, evaluated by the intraclass correlation coefficient (ICC). Blue bars represent short-term reliability and pink bars represent long-term reliability.

We downsampled the datasets to obtain time series with TR of 1.44 s and 2.88 s, and analyzed their synchrony and metastability reliability (Figure 3). The results show that the ICC values of neurodynamic indexes are fairly reproducible in all temporal resolutions. For reliability of synchrony, no significant difference was observed between different temporal resolutions. For the reliability of metastability, the ICC values decreased with TR (For example, in the Destrieux atlas, TR = 0.72s: ICC = 0.5464; TR = 1.44 s: ICC = 0.5335; TR = 2.88 s: ICC = 0.4841).

### 3.3. Effects of Different Spatial Resolution Strategies on Reliabilities of Synchrony and Metastability

**Figure 4:**
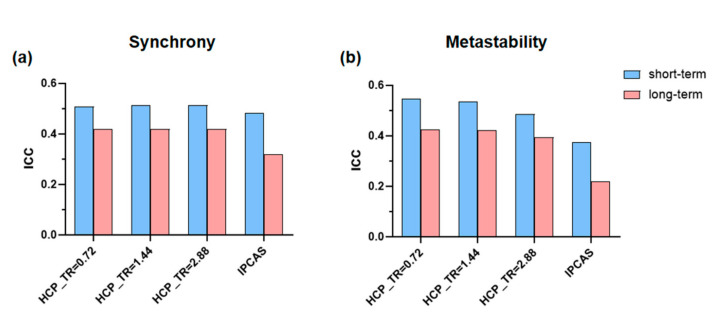
Reliabilities of synchrony (**a**) and metastability (**b**) in different spatial resolutions, evaluated by the intraclass correlation coefficient (ICC). Blue bars represent short-term reliability and pink bars represent long-term reliability.

Since the TR of the HCP dataset is different from that of the IPCAS dataset (HCP: TR = 0.72 s, IPCAS: TR = 2 s), we compare the results after HCP downsampling with IPCAS to illustrate the effect of spatial resolution. It can be seen from Figure 4 that the ICC values of synchrony and metastability of IPCAS are lower than the values of the three groups of HCP. It can be seen from this result that the test–retest reliabilities of synchrony and metastability were affected by the spatial resolution. The higher the spatial resolution, the higher the reliability of synchrony and metastability.

### 3.4. Effects of Denoising Strategies on Reliabilities of Synchrony and Metastability

**Figure 5:**
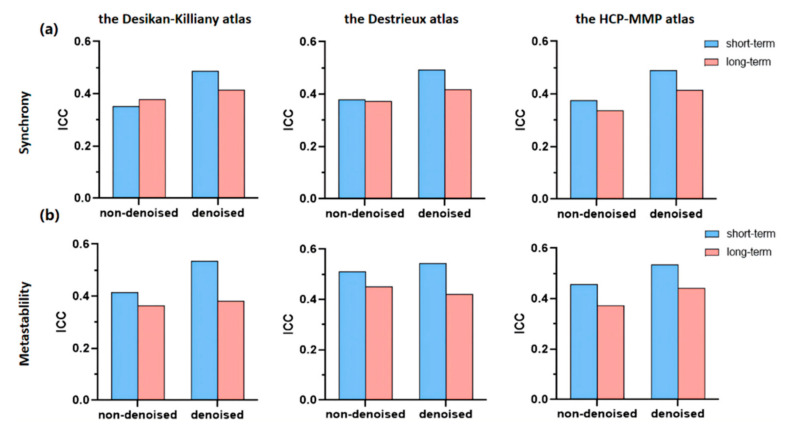
Reliabilities of synchrony (**a**) and metastability (**b**) in denoising strategies for the three brain atlases, evaluated by the intraclass correlation coefficient (ICC). Blue bars represent short-term reliability and pink bars represent long-term reliability.

To investigate the effects of denoising on reliabilities of synchrony and metastability, we used two processing methods for a set of data (3T and 3T-FIX) as controls. The results of the correlational analysis are presented in Figure 5. Among all the brain atlases, synchrony had fair reliability (mean ICC > 0.4) in 3T-FIX but low reliability in 3T (mean ICC < 0.4). Likewise, the same trend as synchrony was found in metastability. For example, based on the HCP-MMP atlas, both synchrony and metastability showed that 3T-FIX was more reliable than 3T (synchrony: ICC_3T-FIX_ = 0.4899, ICC_3T_ = 0.3755; metastability: mean ICC_3T-FIX_ = 0.5342, mean ICC_3T_ = 0.4575). Notably, long-term and short-term retest assessments basically had the same trend in reliabilities between 3T-FIX and 3T. For the long-term reliability of metastability of the Destrieux atlas, although 3T has higher reliability than 3T-FIX, they are all fair. Comparing these results, it can be seen that the test–retest reliabilities of synchrony or metastability were higher based on denoised fMRI analysis than non-denoised fMRI analysis, thus we used denoised datasets for the following studies.

### 3.5. Effects of Different Node Definition Strategies on Reliabilities of Synchrony and Metastability

**Figure 6:**
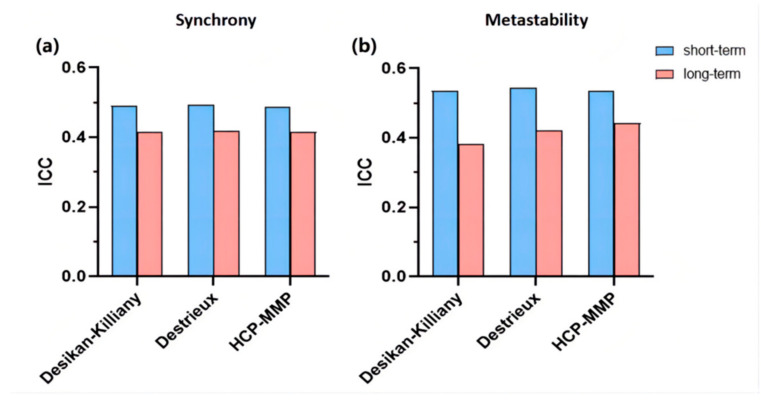
Reliabilities of synchrony (**a**) and metastability (**b**) in different node definition strategies, evaluated by the intraclass correlation coefficient (ICC). Blue bars represent short-term reliability and pink bars represent long-term reliability.

Two indexes were investigated in three brain atlases and compared in our test–retest reliability analysis. As summarized in Figure 6, both synchrony and short-term metastability showed fair reliability (mean ICC > 0.4) based on three brain atlases. Among them, mean ICCs of long-term metastability increase with the number of brain regions (the Desikan-Killiany atlas: ICC = 0.3840; the Destrieux atlas: ICC = 0.4225; the HCP-MMP atlas: ICC = 0.4423).

### 3.6. Reliabilities of Synchrony and Metastability of Different Resting-State Networks

**Figure 7:**
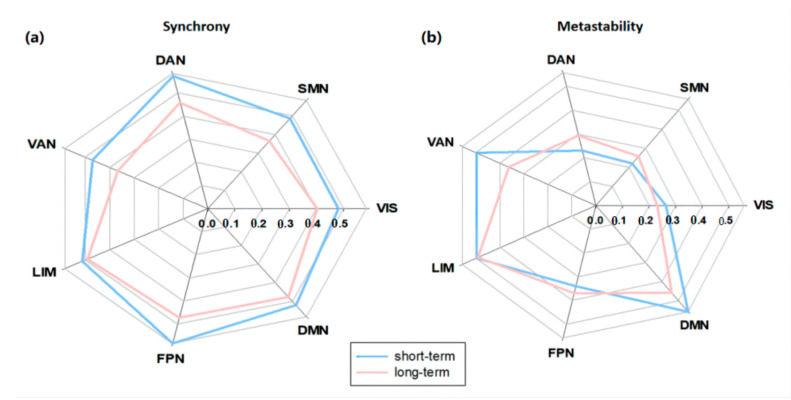
Reliabilities of synchrony (**a**) and metastability (**b**) in different resting-state networks, evaluated by the intraclass correlation coefficient (ICC). Blue lines represent short-term reliability and pink lines represent long-term reliability.

We visualized the values of reliable ICCs for each of those seven networks of the Destrieux atlas for synchrony and metastability as spider graphs in Figure 7. In the short term, the synchrony exhibited ICC values ranging from 0.47 to 0.58, and metastability exhibited ICC values ranging from 0.22 to 0.56; in the long term, the synchrony exhibited ICC values in the range from 0.36 to 0.49, and the metastability exhibited ICC values in the range of 0.23 to 0.49. For synchrony, the highest ICC values were seen in the DMN and FPN, others also had fair reliabilities (mean ICC > 0.4), whereas for metastability, the higher ICC values were seen in the DMN, LIM, and VAN; ICC values of other networks were slightly lower.

**Figure 8:**
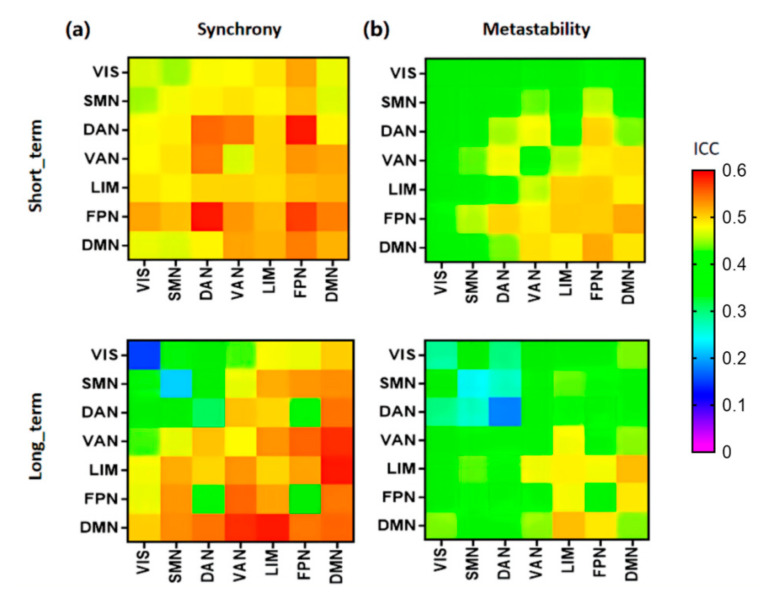
Reliabilities of interaction matrix of synchrony (**a**) and metastability (**b**), evaluated by the intraclass correlation coefficient (ICC). Color bar: 0–0.6.

At the same time, we evaluated the synchrony and metastability interactions between all seven resting-state networks in Figure 8.

Figure 8b shows clearly that the reliabilities of the interaction among VIS, SMN, and DAN had low reliability (ICC < 0.4), and the interaction with other networks had fair reliability (ICC > 0.4). The relatively high ICC values were seen in the interaction with DMN, LIM, and FPN.

## 4. Discussion

One primary goal of this study was to investigate the reliability of neurodynamic indexes (synchrony and metastability) for various data acquisition parameters and data processing methods. Overall, we found that synchrony and metastability showed higher reliability in 7T-FIX data than in 3T-FIX data; and it shows high reliabilities of synchrony and metastability in high spatial resolution; synchrony and metastability of denoising data also have better test–retest reliability. In addition, the research also showed that the reliabilities of synchrony and metastability were less affected by the temporal resolution strategy and the node definition strategy; the short-term reliability is tended to be more stable compared with long-term reliability. In the resting-state networks, DMN and LIM have higher test–retest reliability than others.

### 4.1. High Field Strength, High Temporal resolution, and High Spatial Resolution Are More Reliable in Dynamic Measurement

Overall, for reliabilities of synchrony and metastability, images acquired at 7T-FIX had higher reliability than images acquired at 3T-FIX. These results are in correspondence with the findings of Tak et al. which showed that 7T data have a high reproducibility of effective connectivity [69]. In particular, a higher field strength would enhance the signal-to-noise ratio (SNR), the contrast-to-noise ratio (CNR), and the spatial resolution, as well as it would increase the blood oxygen level dependent (BOLD) effect, making the signal changes in brain function imaging more obvious [45,69,70,71]. Our result suggests that synchrony and metastability achieve fair levels of consistency in describing spontaneous brain activity, and that the reliabilities of synchrony and metastability are better at higher field strengths.

Moreover, this study found that the reliability of synchrony is basically not affected in different temporal resolutions, whereas the reliability of metastability of data of high temporal resolution is higher. The data of high temporal resolution contain more time points, resulting in more information about spontaneous brain activity and potentially higher reproducibility [72,73]. Similarly, consistent with the previous study by Zuo et al., ReHo maps generated from the data of high temporal resolution are substantially more reproducible than those generated from the data of low temporal resolution [74]. This suggests that different temporal resolution strategies influence the neurodynamic indexes, but the effect is not obvious.

Meanwhile, we also found that the reliability of synchrony and metastability of the data of high spatial resolution is higher. Spatial resolution is primarily determined by the volume of the smallest imaging unit (i.e., voxel) and affects the ability of BOLD fMRI data to distinguish activity from distinct functional sub-units such as cortical layers and columns [75]. Image acquisition with higher spatial resolution has higher functional contrast-to-noise (fCNR) and spatial fidelity [75]. There have also been some other studies that found utilizing higher spatial resolution that may allow better quantification of inferior white matter tracts [76]. This suggests that different spatial resolution strategies influence the neurodynamic indexes, and the high spatial resolution may be more suitable for researching them.

### 4.2. Denoising Processing Helps to Improve the Reliability of Dynamic Measurements

We found that the synchrony and metastability of denoising data generally demonstrated fair test–retest reliability. The fMRI signal is affected by many sources of fluctuations, which are collectively referred to as the “noise” components, including effects of motion, non-neuronal physiology, scanner artifacts, and other nuisance sources. This reduces the signal-to-noise ratio, and can mislead statistical analyses attempting to investigate neuronally related brain activation [61,62]. At present, denoising will not alter the information on the neural activity dynamics in the brain [77], suggesting that denoising data may be more suitable for research using neurodynamic indexes.

### 4.3. Nodes Definition Strategies Slightly Affect the Reliability of Dynamic Measurement

We observed a comparable test–retest reliability for the examined strategies of node definition for neurodynamic indexes and found that the reliabilities of the three networks basically remained fair. Obviously, the long-term reliability of metastability shows an upward trend with an increasing number of brain regions. There is speculation that the size of the nodal set may have an influence on the reliability of metastability. There is some evidence that network properties are sensitive to the strategy used to define nodes based on parceling strategies and spatial scales [51,78,79,80,81]. It is noteworthy that the choice of atlas must be approached with some caution, as all of them are valid and important approaches to uncover brain connectivity from different perspectives [82,83,84,85]. Therefore, different strategies used to define nodes will affect the test–retest reliability of neurodynamics indexes, but the effect is not obvious.

### 4.4. DMN and LIM Has Higher Reliability Than Other Networks

Our research results showed that the test–retest reliability of synchrony was fair on almost all resting-state networks. However, across metastability analyses, the reliability values for DMN, LIM, and VAN were relatively stable and relatively higher than those in other networks. The default-model network had a role as a stable core of mental processing [28,86], which is implicated mostly in internal and goal-directed processing [87,88]. In the resting-state environment, it may not correspond to a large amount of information transfer, and the connection information that already existed in the networks was relatively simple [52]. Namely, this could result in the presence of default-mode network with high test–retest reliability. For the visual network and the somatomotor network, low test–retest reliability was an indication of the high dynamics of metastability across time or intra-individual [89].

### 4.5. Short-Term Scanning Interval Can Result in More Reliable and Accurate Values

In this paper, we observed that short-term analyses showed higher test–retest reliability than long-term analyses with synchrony and metastability, and both showed fair reliability. This result agrees with previous findings that the spatial patterns of functional networks were more reliable in short-term scans [90]. It may be explained by the fact that short-term scans may have reduced internal noise with improved reliability estimates and that short-term scanning intervals can result in more stable and accurate values [52,91]. Individual opposite results may be largely attributed to its own poor stability. Our finding here suggests that the scanning interval may affect synchrony and metastability, which needs to be considered in the experimental design of future studies.

## 5. Conclusions

In summary, we examined the stability of synchrony and metastability in the global network and the resting-state networks. Specifically, this study revealed overall fair reliability for these two indexes, and the detected reliabilities were influenced by the strategy used to magnetic flux strengths, temporal resolution, spatial resolution, denoising, define nodes, and scanning interval. In the research of the resting-state networks, it is found that the reliability of synchrony of different resting-state networks was higher, but for the stability of metastability, DMN and LIM were higher than other networks. Our results demonstrated that this systematic exploration of reliability for synchrony and metastability can help to instruct appropriate applications of neural dynamics analysis to discover more information about the brain dynamic neural activity. There are still some limitations in our research. First, the sample size used in our study is small. Although it has reliable and significant results, whether the same phenomenon still exists in a large sample size remains to be studied. Second, in this study, we mainly conducted research on the data of healthy people, and did not analyze the patient data, which can be further studied later.

## Data Availability

Data were provided by the Human Connectome Project, WU-Minn Consortium (HCP; http://www.humanconnectome.org, accessed on 25 December 2021).

## Supplementary Materials

The following are available online at https://www.mdpi.com/article/10.3390/brainsci12010066/s1, Figure S1: Paired sample *t*-test between 3T-FIX and 7T-FIX data analysis; Figure S2: Paired sample *t*-test between different temporal resolution analysis; Figure S3: Paired sample *t*-test between different spatial resolution analysis; Figure S4: Paired sample *t*-test between non-denoised and denoised data analysis; Figure S5: Paired sample *t*-test between different node definition analysis; Figure S6: Paired sample *t*-test between short-term and long-term analysis.
