# Supplementary material for: Test–Retest Reliability of Synchrony and Metastability in Resting State fMRI
Source: Brain Sci. 2021 Dec 31;12(1):66. doi: 10.3390/brainsci12010066 (PMC8773904; doi:10.3390/brainsci12010066)
Supplement: Supplementary file 1 [file brainsci-12-00066-s001.zip › brainsci-1510723-supplementary.pdf]

Supplementary Materials

1. Effects of various influencing factors on reliabilities of synchrony and metastability of resting-state networks

Here, we use the time series extracted from the Destrieux atlas to calculate the influencing factors proposed in the manuscript on the Resting-state networks (RSNs), and perform a statistical analysis of the calculation results. statistical analyses were performed using the statistic toolbox SPSS 19.

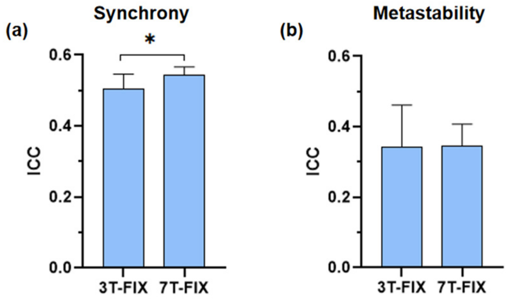

Figure S1. Paired sample t-test between 3T-FIX and 7T-FIX data analysis. The reliability of synchrony exhibited significant differences (*$p < 0.05$).

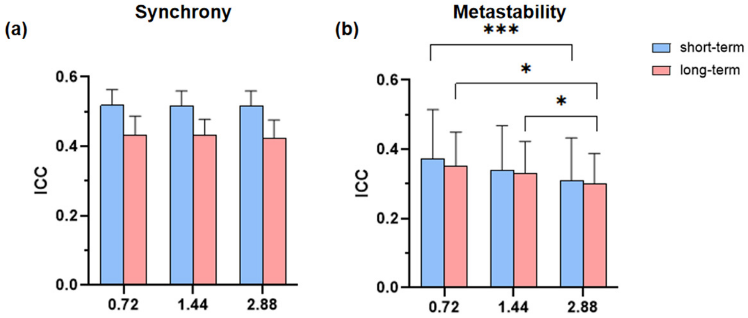

Figure S2. Paired sample t-test between different temporal resolution analysis. The reliability of metastability exhibited significant differences (*$p < 0.05$, ***$p < 0.001$).

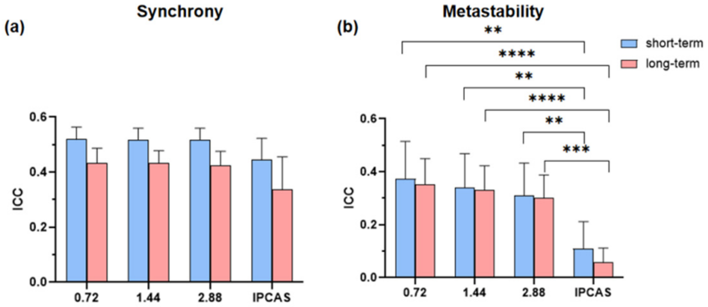

Figure S3. Paired sample t-test between different temporal resolution analysis. The reliability of metastability exhibited significant differences (**$p < 0.01$, ***$p < 0.001$, ****$p < 0.0001$).

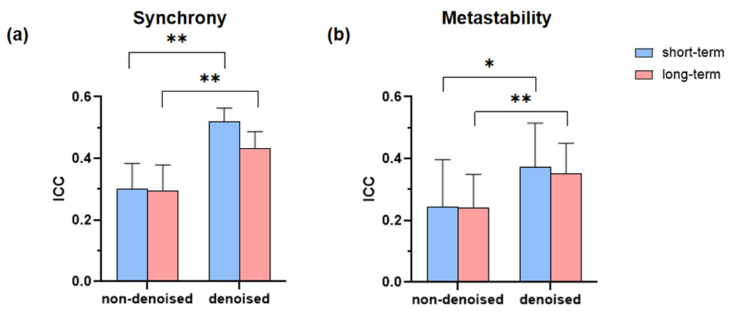

Figure S4. Paired sample t-test between non-denoised and denoised data analysis. The reliability of synchrony and metastability exhibited significant differences (*p < 0.05, **p < 0.01).

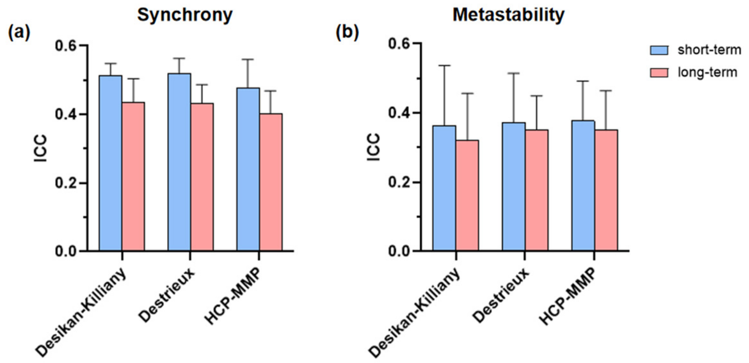

Figure S5. Paired sample t-test between different node definition analysis. The reliability of synchrony and metastability didn't exhibit significant differences.

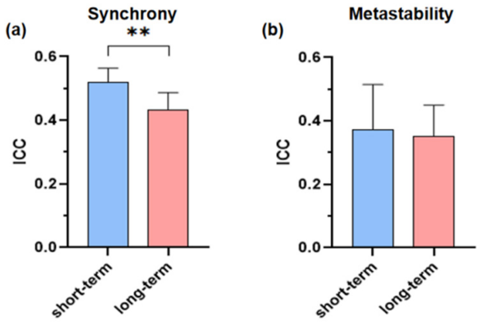

Figure S6. Paired sample t-test between short-term and long-term analysis. The reliability of synchrony exhibited significant differences (**p < 0.01).
